# Identification of *FLT3* and *NPM1* Mutations in Patients with Acute Myeloid Leukaemia

**DOI:** 10.31557/APJCP.2019.20.6.1749

**Published:** 2019

**Authors:** Yuslina Mat Yusoff, Zahidah Abu Seman, Norodiyah Othman, Nor Rizan Kamaluddin, Ezalia Esa, Nor Amalina Zulkiply, Julia Abdullah, Zubaidah Zakaria

**Affiliations:** *Haematology Unit, Cancer Research Centre, Institute for Medical Research, Jalan Pahang, 50588, Wilayah Persekutuan Kuala Lumpur, Malaysia. *

**Keywords:** Acute myeloid leukaemia, FLT3 gene, FLT3, ITD gene, FLT3-D835 gene, NPM1 gene

## Abstract

**Objective:**

The most frequent acquired molecular abnormalities and important prognostic indicators in patients with Acute Myeloid Leukaemia (AML) are fms-like tyrosine kinase-3 gene (*FLT3*) and nucleophosmin-1 (NPM1) mutations. Our study aims to develop a cost effective and comprehensive in-house conventional PCR method for detection of *FLT3*-*ITD*, *FLT3-D835* and *NPM1* mutations and to evaluate the frequency of these mutations in patients with cytogenetically normal (CN) AML in our population.

**Methods:**

A total of 199 samples from AML patients (95 women, 104 men) were included in the study. Mutation analyses were performed using polymerase chain reaction (PCR) and gene sequencing.

**Result:**

Sixty-eight patients were positive for the mutations. *FLT3-ITD* mutations were detected in 32 patients (16.1%), followed by *FLT3-D835* in 5 (2.5%) and NPM1 in 54 (27.1%). Double mutations of NPM1 and *FLT3-ITD *were detected in 23 cases (11.6%). Assays validation were performed using Sanger sequencing and showed 100% concordance with in house method.

**Conclusion:**

The optimized in-house PCR assays for the detection of* FLT3-ITD*, *FLT3-D835* and *NPM1* mutations in AML patients were robust, less labour intensive and cost effective. These assays can be used as diagnostic tools for mutation detection in AML patients since identification of these mutations are important for prognostication and optimization of patient care.

## Introduction

Acute myeloid Leukaemia (AML) is a biologically heterogeneous disease resulting from clonal expansion and loss of differentiation of the haematopoietic progenitor cells (myeloblasts), with complex network of cytogenetic aberrations and molecular mutations (Lowenberg et al., 1999; Estey et al., 2006; Shipley et al., 2009). These genetic markers are the basis for categorization of cases within distinct subgroups and are highly relevant for prediction of prognosis and also for therapeutic decisions in AML (Medinger et al 2017; Torsten et al., 2008). Cytogenetics, age and performance status are well known factors which determine prognosis and therapy. It has long been appreciated that cytogenetic factors are independent predictors for the prognosis of AML patients (Schiffer and Stone, 2003). However, for 50% of AML patients, no cytogenetic markers can be found (Döhner et al., 2010; Schlenk et al., 2008; Grimwade et al., 2010; Mrozek et al., 2004). Genetic mutations that escape cytogenetic detection have increasingly been discovered, and these mutations may serve as potential markers for prognostication in AML and can be used as molecular targets for precision medicine (Foran, 2010).

Fms-like tyrosine kinase-3 gene (*FLT3*) and nucleophosmin-1 (NPM1) mutations are the most frequent acquired molecular abnormalities in cytogenetically normal (CN) AML (Falini et al., 2005; Renneville et al., 2008). Many studies have shown that *FLT3* gene plays an important role in the proliferation, differentiation and survival of multipotent stem cells. This gene is located at chromosome 13q12 and it encodes for a membrane bound receptor tyrosine kinase and was expressed normally by myeloid and lymphoid progenitor cells. It consists of five immunoglobulin-like extracellular domains that further divides into a single transmembrane domain, a juxtamembrane domain (JMD) and intracellular domains. Mutation of these genes causes poor prognosis in both adult and childhood AML. Two major types of *FLT3* mutations are found among AML patients namely the internal tandem duplication (ITD) and point mutation in tyrosine kinase domain (D835) (Levis, 2013). *FLT3-ITD* involves insertion of 6 to 180 nucleotides into exon 11 and exon 12 in the juxtamembrane region (JM) (Markovic et al., 2005), which always lead to in frame transcript (Hayakawa et al., 2000). Meanwhile, *FLT3-D835* involves missense point mutation at codon 835 which alters the aspartic acid residue and causes constitutive activation of *FLT3* receptor (Abu-Duhier et al., 2001). These mutations make up for 20 to 37% (Chirayu et al., 2005; Wang et al., 2005) and 7.7% (Yamamoto et al., 2001) of total AML respectively.

Nucleoplasmin (NPM1) is a phosphoprotein that shuttles continuously between the nucleus and cytoplasm and is involved in regulation of the ARF-p53-tumor suppressor pathway (Borer et al., 1989). *NPM1* gene is located on 5q35.1 and contains 12 exons (Morris et al., 1994). Mutation in this gene leads to aberrant cytoplasmic localization of nucleoplasmin protein (Falini et al., 2005). *NPM1* mutations are present in 50% to 60% of patients with CN-AML and 30% to 35% of all AML cases (Verhaak et al., 2005; Ivey et al., 2016). More than 95% of the NPM1 mutations consist of a 4–base pair (bp) insertion at position 863. The most frequently occurring have been named type A (c.860_863dupTCTG), which accounts for 69% to 80% of adults cases, followed by type B (c.863_864insCATG) which was found in 10% to 11% cases and type D (c.863_864insCCTG) in about 5% to 10%. The remaining types of mutations account for 10% to 12% of cases (Falini et al., 2005; Alpermann et al., 2016). There are at least 20 types of NPM1 mutations with 4-bp insertions (including type C) at the same position, and a minority of insertions at other locations in exon 12. (Falini et al., 2005; Thiede et al., 2006).

Early identification of *FLT3* and *NPM1* gene mutations helps clinician to provide up-front treatment regimens and therapeutic strategies for the AML patients. There are several commercially available kits for *FLT3* and *NPM1 *gene mutations, however the available kits are relatively expensive. Therefore, there is a need to develop a simple, rapid, reliable and cost-effective test in the diagnostic testing. Our study aims to establish a cost effective and rapid polymerase chain reaction (PCR) method for routine diagnostic protocols. We also present the incidence of *FLT3-ITD*, *FLT3-D835* and *NPM1* mutations of CN-AML patients in our population.

## Materials and Methods

*Samples and DNA Preparation*

One hundred and ninety-nine CN-AML patients were included in this study. *FLT3-ITD*, *FLT3-D835* and *NPM1 *mutations testing were performed on peripheral blood and bone marrow aspirates samples. All samples were collected in accordance to the Ethics of Human research guidelines and DNA was extracted using the QIAmp DNA blood mini kit according to the manufacturer’s instruction (Qiagen, Hilden, Germany). 

*PCR amplification*

For mutational analysis of *FLT3-ITD*, *FLT3-D835* and *NPM1*, genomic DNA of AML patients were amplified using primer sets as described previously (Dunna et al., 2010; Xu et al., 2005). The primers were synthesized by First BASE Laboratories Sdn Bhd, Singapore.

Primers for both assays were designed based on *FLT3* gene which encodes a class III receptor tyrosine kinase that regulates haematopoiesis (Accession No: NM_004119.2). *FLT3-ITD* primers were designed between exon 14 and 15 of the *FLT3* gene, with the amplified product size of 329 bp, while *FLT3-D835* primers were designed for exon 20 of the *FLT3* gene, which produced 114 bp products. *NPM1* primers were designed to amplify a product size that contains the coding region of *NPM1* exon 12 (Accession No: NM_002520).

*FLT3-ITD* PCR: Since *FLT3-ITD* mutation was located within exon 14 and 15, PCR amplification was performed using 5’- GCA ATT TAG GTA TGA AAG CCA GC -3’ (ITD_14F) and 5’- CTT TCA GCA TTT TGA CGG CAA CC -3’ (ITD_15R) primers. PCR was performed in a total volume 25 µl of 1X Maxima Hot Start Taq buffer, 3 mM MgCl2, 0.2 mM dNTPs, 0.8 µM of ITD_14F and ITD_15R primers, 1.25 U Maxima Hot Start Taq polymerase and 50-100ng/µl of genomic DNA. The reaction was carried out through initial denaturation at 95^o^C for 7 minutes, followed by 35 cycles of denaturation at 94^o^C for 1 minute, annealing at 60^o^C for 45 seconds and extension at 72^o^C for 1 minute, and final extension at 72^o^C for 7 minutes. 

*FLT3-D835* PCR: Primers used for the D835 were 5’-CCG CCA GGA ACG TGC TTG-3’ (D835_F) and 5’-GCA GCC TCA CAT TGC CCC-3’ (D835_R). PCR was performed in a total volume 25 µl of 1X Maxima Hot Start Taq buffer, 2 mM Q-solution, 3 mM MgCl_2_, 0.2 mM dNTPs, 0.8 µM of D835_F and D835_R primers, 1.25 U Maxima Hot Start Taq polymerase and 50-100ng/µl of genomic DNA. Reaction was carried out through initial denaturation at 95^o^C for 9 minutes, followed by 35 cycles of denaturation at 94^o^C for 30 seconds, annealing at 59^o^C for 1 minute and extension at 72^o^C for 1 minute, and final extension at 72^o^C for 10 minutes. After amplification, 10 µl of PCR product was digested with 1 U EcoRV enzyme and 2X digestion buffer. Digestion reaction was performed at 37^o^C for 15 minutes, followed by enzyme deactivation at 80^o^C for 20 minutes. PCR product for *FLT3-ITD* and *FLT3-D835* were then visualized via Agilent Bioanalyzer 2100 System plus DNA 1000 LabChip kit (Agilent Technologies, CA, USA). 

NPM1 PCR: Genomic DNA was amplified with 5’- TTA ACT CTC TGG TGG TAG AAT GAA-3’ (NPM1_F) and 5’- TGT TAC AGA AAT GAA ATA AGA CGG-3’ (NPM1_R) primers. PCR was performed in a total volume 20 µl of 1X Maxima Hot Start Taq buffer, 2.5 mM MgCl2, 0.7 µM of NPM1_F and NPM1_R primers, 1.25 U Maxima Hot Start Taq polymerase and 50-100ng/µl of genomic DNA. After an initial denaturation at 94°C for 3 minutes, DNA was amplified in 35 cycles at 95°C for 1 minute, 60°C for 45 seconds, 72°C for 2 minutes, followed by a final extension at 72°C for 7 minutes. PCR products were visualized under UV light after electrophoresis of 5 μl product in 4% agarose gel.

## Results

**Figure 1:**
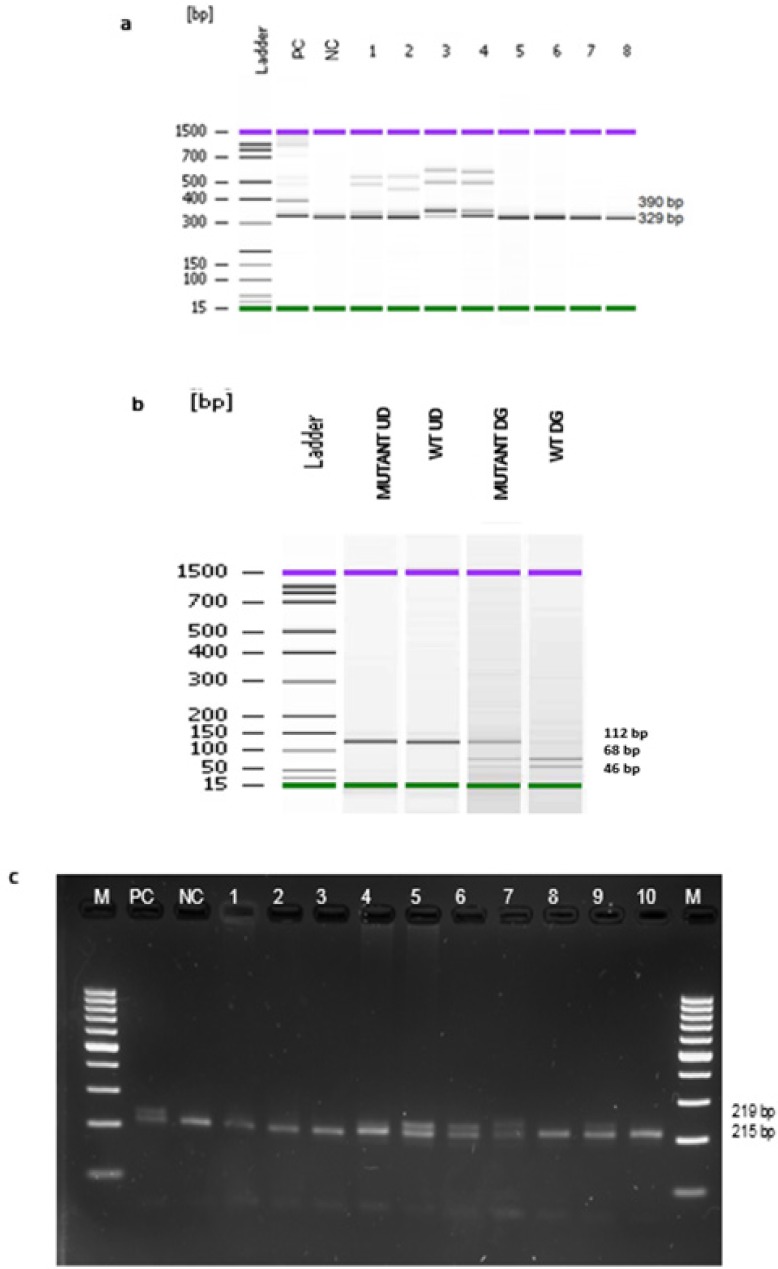
(a), PCR amplification results for *FLT3-ITD* gene mutation. Ladder: DNA marker (100 bp ladder), PC: Positive control, NC: negative control, Lane 1-4: Positive result, Lane 5-8: Negative result; (b), PCR amplification results for *FLT3-D835* gene mutation. Ladder: DNA marker (100 bp ladder), Mutant UD: Mutant (Undigested), WT UD: wild type (Undigested), Mutant DG: Mutant (Digested), WT DG: wild type (Digested); (c), PCR amplification results for NPM1 gene mutation. Lane M: DNA marker (100 bp ladder), Lane PC: Positive control, Lane NC: Negative control, Lane 4 - 7, 9: Positive result, Lane 1-3, 8, 10: Negative result

PCR amplicon with ITD gene mutation showed different sizes of PCR products (≥ 329 bp) due to tandem repeat mutation which occurs at variable locations and different lengths in the *ITD* gene. Negative result produced 329 bp band which indicates absence of *FLT3-ITD* mutation, while positive result produced bands larger than 329 bp which indicate presence of *FLT3-ITD* mutation. It has also been confirmed that the size of the* ITD* was always a multiple of three bases (Mills et al, 2005) (Figure 1 (a)). 

Amplicon of *FLT3-D835* gene was amplified and digested using EcoRV enzyme (GATATC) recognized position at 835 to produce 68 bp and 46 bp products. Digestion step is crucial to differentiate between normal and mutated samples. *FLT3-D835* mutation changes the G to T base and modified the binding site of EcoRV. Thus, EcoRV cannot recognise the amplicon sequence and produced 112 bp of PCR product (Figure 1 (b)). 

Genomic fragment of *NPM1* produced 215 bp and 219 bp for mutant NPM1 and 215 bp for wild-type *NPM1 *(Figure 1 (c)). 

**Figure 2:**
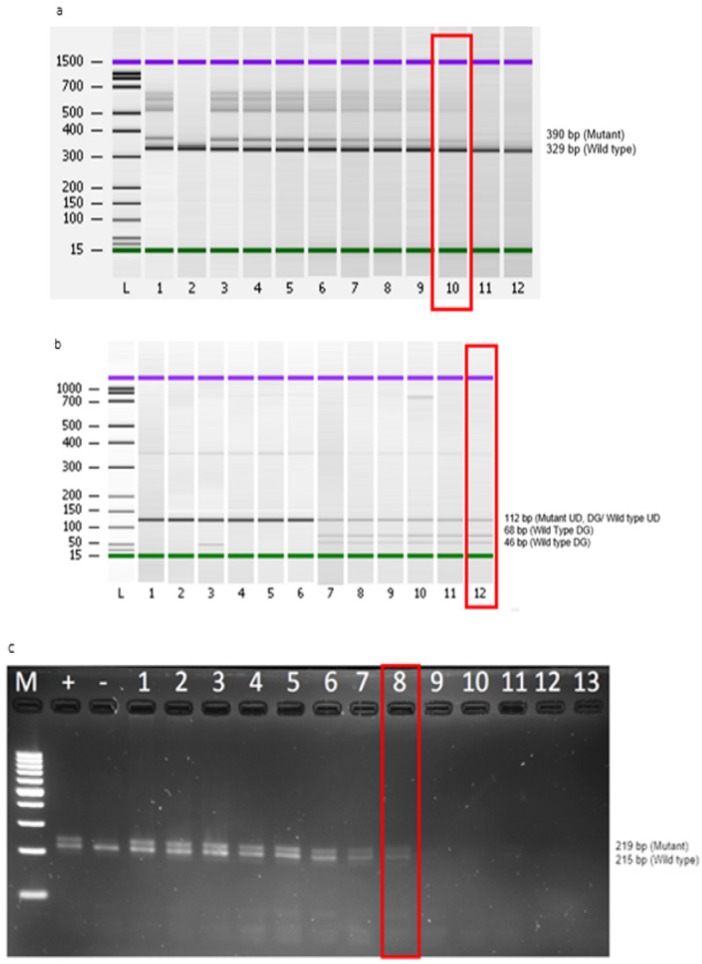
(a), Testing limit of detection on *FLT3-ITD* primers. L: DNA marker (100 bp ladder), 1: Positive control (PC), 2: No template control (NTC), 3: 50 ng, 4: 25 ng, 5: 12.5 ng, 6: 6.25 ng, 7: 3.13 ng, 8: 1.56 ng, 9: 0.78 ng, 10: 0.40 ng, 11: 0.20 ng, 12: 0.10 ng; (b), Testing for detection limitations of *FLT3-D835* primers. L: DNA marker (100 bp ladder), 1: 50 ng, 2: 25 ng, 3: 12.5 ng, 4: 6.25 ng, 5: 3.13 ng, 6: 1.56 ng, 7: 50 ng, 8: 25 ng, 9: 12.5 ng, 10: 6.25 ng, 11: 3.13 ng, 12: 1.56 ng; 1-6: Undigested samples, 7-12: Digested samples; (c), Testing for detection limitations of NPM1 primers. M: DNA marker (100 bp ladder), Lane +: Positive control (PC), Lane -: Negative control (NC), 1: 200 ng, 2: 100 ng, 3: 50 ng, 4: 25 ng, 5: 12.5 ng, 6: 6.25 ng, 7: 3.13 ng, 8: 1.56 ng, 9: 0.78 ng, 10: 0.39 ng, 11: 0.19 ng, 12: 0.09 ng, 13: 0.04 ng

Evaluation of limit of detection for *FLT3-ITD*, *FLT3-D835* and *NPM1* primers were performed by serial dilution of DNA. DNA concentration limitation for detection of *FLT3-ITD* and *FLT3-D835* primers were 0.4 ng and 1.56 ng respectively using Agilent 2100 Bioanalyzer (Figure 2 (a,b)). DNA fragment of *NPM1 *primer, using PCR-agarose gel electrophoresis could detect 1.56 ng of the DNA concentration (Figure 2 (c)).

A total of 199 patients (104 men, 95 women) with *CN-AML* were included in this study. Their demographic features including age, race and gender was summarized as in Table 1. Mutations were detected in 68 patients (34.2%), single mutation for *FLT3-ITD* was detected in 32 patients (16.1%), *FLT3-D835* in 5 (2.5%) and *NPM1 *in 54 (27.1%), while double mutations of *NPM1* and *FLT3-ITD* were detected in 23 (11.6%). Assays validation were performed on all PCR-positive and 30 PCR-negative samples using Sanger sequencing, which showed 100% conformity with *FLT3-ITD*, *FLT3-D835* and *NPM1 *mutation genes.

Clinical characteristics of CN-AML patients were analysed by comparing these four groups (Table 2). Haemoglobin (Hb) level in the *FLT3-ITD* and *NPM1 *mutations was significantly lower as compared to the group without mutations. Clinical parameters of the groups with *FLT3-D835 *and those who had both *FLT3-ITD* and *NPM1* positivity were not significantly different from group without mutations, probably due to the smaller number of patients. 

**Table 1:** Demographic Data and Types of Mutations in CN-AML Patients

Parameter	All CN-AML patients n = 199	CN-AML without mutation n = 131	CN-AML with mutations			
			*FLT3-ITD* n = 32	*FLT3-D835* n = 5	*NPM1* n = 54	*FLT3-ITD *and *NPM1* n = 23
Gender, N (%)						
Male	104 (52.3)	77 (58.8)	14 (43.8)	2 (40.0)	20 (37.0)	9 (39.1)
Female	95 (47.7)	54 (41.2)	18(56.3)	3 (60.0)	34 (63.0)	14 (60.9)
Race, N (%)						
Malay	102 (51.3)	64 (48.9)	19 (59.4)	3 (60.0)	28 (51.9)	12 (54.5)
Chinese	57 (28.6)	43 (32.8)	4 (12.5)	0 (0.0)	13 (24.1)	3 (13.6)
Indian	12 (6.0)	8 (6.1)	3 (9.4)	0 (0.0)	4 (7.4)	3 (13.6)
Others	28 (14.1)	16 (12.2)	6 (18.8)	2 (40.0)	9 (16.7)	5 (18.2)
Age						
Mean years (range)	46.16 (16-83)	44.99 (16-83)	48.28 (18-71)	51 (21-68)	49.31 (18-68)	50.73 (18-67)

**Table 2:** Comparison of Clinical Parameters among Mutated and Non-Mutated Groups

Clinical characteristics	All CN-AML patients n = 199	CN-AML without mutations n = 131	CN-AML with mutations			
			*FLT3-ITD* n = 32	*FLT3-D835* n = 5	*NPM1* n = 54	*FLT3-ITD & NPM1* n = 23
Median Hb level, g/dl (range)	8.83 (1.1-18.5)	9.06 (1.1-18.5)	8.49 (4.0-13.5)	8.52 (7.1-9.8)	8.46 (6.2-13.5)	8.68 (6.3-13.5)
			p = 0.391	p = 0.772	p = 0.094	p = 0.759
Median WBC count, 1 X 10^9^/L (range)	48.18 (1.8-150)	47.28 (1.8-150)	53.356 (6.1-150)	87.72 (5.3-150)	45.5 (4.3-150)	54.00 (6.1-150)
			p = 0.487	p = 0.274	p = 0.961	p = 0.530
Median platelet count, 1 X 10^9^/L (range)	143.70 (15.2-736)	159.46 (15.2-736)	112.53 (16-368)	193.6 (105-335)	111.04 (20-390)	124.82 (54-368)
			p = 0.024	p = 0.376	p = 0.006	p = 0.273

## Discussion

FLT3 and NPM1 gene mutations testing by commercialized kits are relatively expensive. This has led us to develop a simple, reliable and cost effective in-house conventional PCR method for rapid detection of these mutations. CN-AML patients may benefit from *FLT3* and *NPM1* gene mutation testings as it provides useful information on patient prognosis and assist clinical decision for personalised treatment. During the lasts decades, a wide variety of methods have been developed for detection of *FLT3* and *NPM1* mutations such as genomic DNA sequencing of different mutation-specific RT-PCR assays, denaturing high-performance liquid chromatography, capillary electrophoresis, allele-specific oligonucleotide polymerase chain reaction (ASO-PCR) and high-resolution melting analysis (Ammatuna et al., 2005; Chopra et al., 2016; Lu et al., 2012; Noguera et al., 2005; Ottone et al., 2008; Tan et al., 2008). 

Commercial kit-based assays were used because they are reproducible, validated and certified. Procedures for commercialized kits are also less labour intensive, does not require in-depth molecular biology skills and experience as required for in-house assay development. However, commercial kit-based assays incur a higher cost to procure reagents and equipment since there is a lack of universal compatibility with real-time PCR platforms. Conventional PCR assay has been widely used as a diagnostic tool since it is simple, more cost-effective. Development of in-house assays are beneficial in term of platform compatibility and relatively more economical as compared to kits, although the assays are certainly more labour intensive specifically at the standardisation stages which require rigorous validation protocol.

*FLT3* gene mutation has negative impact on adult and childhood AML (Hollink et al., 2009; Kiyoi et al., 1999). This gene is important for cell survival, proliferation and differentiation of haematopoietic cells. Thus, mutation on this gene may disturb the balance between cell proliferation and differentiation. *FLT3* gene mutation causes the activation of tyrosine kinase receptor which causes multiple downstream signalling pathways and leads to leukaemogenesis (Grafone et al., 2012). 

*FLT3* gene mutations are found in 25 to 45% among AML patients and *FLT3-ITD* mutations are found in approximately 20 to 37% (Auewarakul et al., 2005; Burnatt et al., 2017). *FLT3-D835* mutations are found to be less common as compared to *FLT3-ITD *mutations with the percentage of 5.8 to 7.7%. NPM1 mutations at exon 12 are the most common genetic abnormality in AML, found in approximately 24 to 45% of all AML cases and up to 60% of adult CN-AML (Falini et al., 2005; Döhner et al., 2005; Weina et al., 2006).

From the total of 199 CN-AML patients in this study,* FLT3-ITD*, *FLT3-D835 *and *NPM1* mutations were detected in 16.1% (32/199), 2.5% (5/199), and 27.1% (54/199) respectively. These findings are lower than reported among CN-AML in German (31 to 32% *FLT3-ITD*, 11 to 14% *FLT3-D835* , 45.7 to 53% *NPM1*) (Frohling et al., 2002; Schlenk et al., 2008; Thiede et al., 2006), United States (34.6% *FLT3-ITD*, 7.4% *FLT3-D835*) (Whitman et al., 2008), United Kingdom (34% *FLT3-ITD*, 62% *NPM1*) (Gale et al., 2008) and Japan (47.4% *NPM1*) (Suzuki et al., 2005). Frequency of FLT3-ITD and FLT3-D835 in our study was comparable to a preliminary study conducted by (Dehbi et al., 2013) in CN-AML patients (18.2%* FLT3-ITD*, 3% *FLT3-D835*). Previous study reported that mutations of *FLT3-ITD* in adults are higher than children. This finding may partially explain a poorer prognosis in adult than childhood *AML* (Frohling et al., 2002).

Identification of *FLT3* and* NPM1* gene mutations might help clinician to provide proper treatment for the patients. In this study, PCR assays for the detection of *FLT3-ITD*, *FLT3-D835* and* NPM1 *were successfully developed. The use of bioanalyzer as detection method for PCR amplicons reduces the workload as it involves simple procedures. Sanger sequencing was performed for validation of these assays. Analytically, the developed *FLT3-ITD*, *FLT3-D835* and *NPM1 PCR* assays have lower limit of detection of 0.4 ng, 1.56 ng and 1.56 ng respectively, which was slightly better compared to other study (Liu et al., 2015). Our in-house PCR assays are reliable, requires small volume of samples, less labour intensive with shorter turnaround time and cost effective.

In conclusion identification of *FLT3-ITD*, *FLT3-D835 *and *NPM1* mutations in CN-AML are important for prognostication, treatment decision and optimization of patient care. Therefore, the use of these PCR assays as diagnostic tool is a practical approach for mutation detection in *AML* as it is simple, robust and cost effective. 

## References

[B1] Abu-Duhier FM, Goodeve AC, Wilson GA (2001). Identification of novel FLT-3 Asp835 mutations in adult acute myeloid leukaemia. Br J Haematol.

[B2] Alpermann T, Schnittger S, Eder C (2016). Molecular subtypes of NPM1 mutations have different clinical profiles, specific patterns of accompanying molecular mutations and varying outcomes in intermediate risk acute myeloid leukemia. Haematologica.

[B3] Ammatuna E, Noguera NI, Zangrilli D (2005). Rapid detection of nucleophosmin (NPM1) mutations in acute myeloid leukemia by denaturing HPLC. Clin Chem.

[B4] Auewarakul CU, Sritana N, Limwongse C (2005). Mutations of the FLT3 gene in adult acute myeloid leukemia: determination of incidence and identification of a novel mutation in a Thai population. Cancer Genet Cytogenet.

[B5] Borer RA, Lehner CF, Eppenberger HM (1989). Major nucleolar proteins shuttle between nucleus and cytoplasm. Cell.

[B6] Burnatt G, Licínio MA, Gaspar PC (2017). Analysis of the presence of FLT3 gene mutation and association with prognostic factors in adult and pediatric acute leukemia patients. Braz J Pharm Sci.

[B7] Chirayu UA, Narongrit S, Chanin L (2005). Mutation of FLT3 gene in adult acute myeloid leukemia: determination of incidence and identification of a novel mutation in a Thai population. Cancer Genet Cytogenet.

[B8] Chopra A, Soni S, Pati H (2016). Nucleophosmin mutation analysis in acute myeloid leukaemia: Immunohistochemistry as a surrogate for molecular techniques. Indian J Med Res.

[B9] Dehbi H, Kassogue Y, Nasserddine S (2013). FLT3-ITD Incidence and FLT-D835 Mutations in Acute Myeloid Leukemia Patients with Normal Karyotype in Morocco: A Preliminary Study. Middle East J Cancer.

[B10] Dohner H, Estey EH, Amadori S (2010). Diagnosis and management of acute myeloid leukemia in adults: recommendations from an international expert panel, on behalf of the European LeukemiaNet. Blood.

[B11] Dunna NR, Rajappa S, Digumarti R (2010). Fms like tyrosine kinase (FLT3) and nucleophosmin 1 (NPM1) mutations in de novo normal karyotype acute myeloid leukemia (AML). Asian Pac J Cancer Prev.

[B12] Estey E, Dohner H (2006). Acute myeloid leukaemia. Lancet.

[B13] Falini B, Mecucci C, Tiacci E (2005). Cytoplasmic nucleophosmin in acute myelogenous leukemia with a normal karyotype. N Engl J Med.

[B14] Frohling S, Schlenk RF, Breitruck J (2002). Prognostic significance of activating FLT3 mutations in younger adults (16 to 60 years) with acute myeloid leukemia and normal cytogenetics: a study of the AML Study Group Ulm. Blood.

[B15] Gale RE, Green C, Allen C (2008). The impact of FLT3 internal tandem duplication mutant level, number, size, and interaction with NPM1 mutations in a large cohort of young adult patients with acute myeloid leukemia. Blood.

[B16] Grafone T, Palmisano M, Nicci C (2012). An overview on the role of FLT3-tyrosine kinase receptor in acute myeloid leukemia: biology and treatment. Oncol Rev.

[B17] Grimwade D, Hills RK, Moorman AV (2010). Refinement of cytogenetic classification in acute myeloid leukemia: determination of prognostic significance of rare recurring chromosomal abnormalities among 5876 younger adult patients treated in the United Kingdom Medical Research Council trials. Blood.

[B18] Hayakawa F, Towatari M, Kiyoi H (2000). Tandem-duplicated Flt3 constitutively activates STAT5 and MAP kinase and introduces autonomous cell growth in IL-3-dependent cell lines. Oncogene.

[B19] Hollink IH, Zwaan CM, Zimmermann M (2009). Favorable prognostic impact of NPM1 gene mutations in childhood acute myeloid leukemia, with emphasis on cytogenetically normal AML. Leukemia.

[B20] Ivey A, Hills RK D Phil, Simpson MA (2016). Assessment of minimal residual disease in standard-risk AML. N Engl J Med.

[B21] Kiyoi H, Naoe T, Nakano Y (1999). Prognostic implication of FLT3 and N-RAS gene mutations in acute myeloid leukemia. Blood.

[B22] Levis M (2013). FLT3 mutations in acute myeloid leukemia: what is the best approach in 2013?. Hematol Am Soc Hematol Educ Program.

[B23] Liu HE, Ko C-H, Lam F (2015). Establishment of a cost-effective method to detect FLT-ITD and D835 mutations in acute myeloid leukaemia patients in the Taiwanese population. Tzu Chi Med J.

[B24] Lowenberg B, Downing JR, Burnett A (1999). Acute myeloid leukemia. N Engl J Med.

[B25] Lu Y, Wang Q, Mu QT (2012). Establishment of a rapid and easy method for simultaneous detection of FLT3-ITD and NPM1 gene mutations in acute myeloid leukemia. Zhonghua Yi Xue Yi Chuan Xue Za Zhi.

[B26] Markovic A, MacKenzie KL, Lock RB (2005). FLT-3: a new focus in the understanding of acute leukemia. Int J Biochem Cell Biol.

[B27] Mrozek K, Heerema NA, Bloomfield CD (2004). Cytogenetics in acute leukemia. Blood Rev.

[B28] Morris SW, Kirstein MN, Valentine MB (1994). Fusion of a kinase gene, ALK, to a nucleolar protein gene, NPM, in non-Hodgkin’s lymphoma. Science (New York NY).

[B29] Noguera NI, Ammatuna E, Zangrilli D (2005). Simultaneous detection of NPM1 and FLT3-ITD mutations by capillary electrophoresis in acute myeloid leukemia. Leukemia.

[B30] Ottone T, Ammatuna E, Lavorgna S (2008). An allele-specific RT-PCR assay to detect type a mutation of the Nucleophosmin-1 gene in acute myeloid leukemia. J Mol Diagn.

[B31] Renneville A, Roumier C, Biggio V (2008). Cooperating gene mutations in acute myeloid leukemia: a review of the literature. Leukemia.

[B32] Schlenk RF, Dohner K, Krauter J (2008). Mutations and treatment outcome in cytogenetically normal acute myeloid leukemia. N Engl J Med.

[B33] Shipley JL, Butera JN (2009). Acute myelogenous leukemia. Exp Hematol.

[B34] Suzuki T, Kiyoi H, Ozeki K (2005). Clinical characteristics and prognostic implications of NPM1 mutations in acute myeloid leukemia. Blood.

[B35] Tan AY, Westerman DA, Carney DA (2008). Detection of NPM1 exon 12 mutations and FLT3 - internal tandem duplications by high resolution melting analysis in normal karyotype acute myeloid leukemia. J Hematol Oncol.

[B36] Thiede C, Koch S, Creutzig E (2006). Prevalence and prognostic impact of NPM1 mutations in 1485 adult patients with acute myeloid leukemia (AML). Blood.

[B37] Verhaak RG, Goudswaard CS, van Putten W (2005). Mutations in nucleophosmin (NPM1) in acute myeloid leukemia (AML): association with other gene abnormalities and previously established gene expression signatures and their favorable prognostic significance. Blood.

[B38] Wang L, Lin D, Zhang X (2005). Analysis of FLT3 internal tandem duplication and D835 mutations in Chinese acute leukemia patients. Leuk Res.

[B39] Whitman SP, Ruppert AS, Radmacher MD (2008). FLT3 D835/I836 mutations are associated with poor disease-free survival and a distinct gene-expression signature among younger adults with de novo cytogenetically normal acute myeloid leukemia lacking FLT3 internal tandem duplications. Blood.

[B40] Xu B, Li L, Tang JH (2005). Detection of FLT3 gene and FLT3/ITD mutation by polymerase chain reaction-single-strand conformation polymorphism in patients with acute lymphoblastic leukemia. Di Yi Jun Yi Da Xue Xue Bao.

[B41] Yamamoto Y, Kiyoi H, Nakano Y (2001). Activating mutation of D835 within the activation loop of FLT3 in human hematologic malignancies. Blood.

